# Aqua­chloridodimethyl­phenyltin(IV)–15-crown-5 (2/1)

**DOI:** 10.1107/S1600536808011070

**Published:** 2008-04-26

**Authors:** Mahsa Armaghan, Mostafa M. Amini, Amirreza Azadmehr, Shan Gao, Seik Weng Ng

**Affiliations:** a Department of Chemistry, Shahid Beheshti University, Tehran, Iran; b School of Chemistry and Materials Science, Heilongjiang University, Harbin 150080, People’s Republic of China; c Department of Chemistry, University of Malaya, 50603 Kuala Lumpur, Malaysia

## Abstract

The Sn^IV^ atom in the title compound, 2[Sn(CH_3_)_2_(C_6_H_5_)Cl(H_2_O)]·C_10_H_20_O_5_, exists in a *trans-*C_3_SnClO trigonal bipyramidal geometry in which the organo substituents occupy the equatorial sites. The coordinated water mol­ecule forms two hydrogen bonds to the 15-crown-5 mol­ecule, which is disordered about a center of inversion.

## Related literature

For ‘outer-sphere coordination’ organotin complexes with 15-crown-5, see: Amini *et al.* (1994 ▶); Chee *et al.* (2003 ▶); Yap *et al.* (1996 ▶). For the refinement of 15-crown-5 type crystal structures that are disordered about a center of inversion, see: Ng (2005 ▶). For the analogous adduct of SnCl(CH_3_)_2_(C_6_H_5_)(H_2_O) with 18-crown-6, see: Amini *et al.* (2002 ▶).
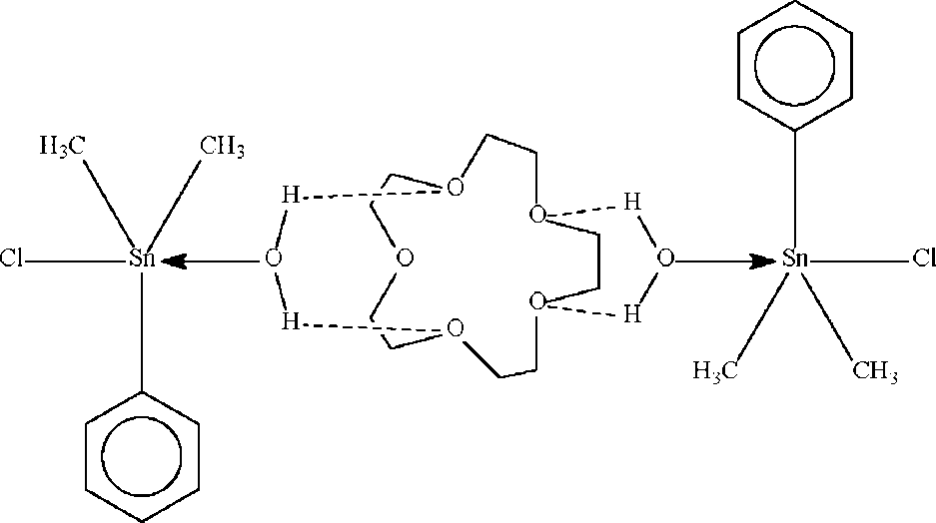


## Experimental

### 

#### Crystal data

2[Sn(CH_3_)_2_(C_6_H_5_)Cl(H_2_O)]·C_10_H_20_O_5_
*M* *_r_* = 778.91
Monoclinic, 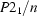

*a* = 9.8700 (3) Å
*b* = 18.9814 (5) Å
*c* = 9.8770 (3) Å
β = 113.636 (1)°
*V* = 1695.2 (1) Å^3^
*Z* = 2
Mo *K*α radiation
μ = 1.67 mm^−1^
*T* = 295 (2) K
0.30 × 0.25 × 0.20 mm

#### Data collection

Rigaku R-AXIS RAPID diffractometer
Absorption correction: multi-scan (*ABSCOR*; Higashi, 1995 ▶) *T*_min_ = 0.534, *T*_max_ = 0.732
16379 measured reflections
3869 independent reflections
3433 reflections with *I* > 2σ(*I*)
*R*_int_ = 0.023

#### Refinement

*R*[*F* ^2^ > 2σ(*F* ^2^)] = 0.042
*wR*(*F* ^2^) = 0.128
*S* = 1.06
3869 reflections
159 parameters
30 restraints
H-atom parameters constrained
Δρ_max_ = 1.29 e Å^−3^
Δρ_min_ = −1.14 e Å^−3^

### 

Data collection: *RAPID-AUTO* (Rigaku, 1998 ▶); cell refinement: *RAPID-AUTO*; data reduction: *CrystalStructure* (Rigaku/MSC, 2002 ▶); program(s) used to solve structure: *SHELXS97* (Sheldrick, 2008 ▶); program(s) used to refine structure: *SHELXL97* (Sheldrick, 2008 ▶); molecular graphics: *X-SEED* (Barbour, 2001 ▶); software used to prepare material for publication: *publCIF* (Westrip, 2008 ▶).

## Supplementary Material

Crystal structure: contains datablocks global, I. DOI: 10.1107/S1600536808011070/tk2247sup1.cif

Structure factors: contains datablocks I. DOI: 10.1107/S1600536808011070/tk2247Isup2.hkl

Additional supplementary materials:  crystallographic information; 3D view; checkCIF report

## Figures and Tables

**Table:** 

Sn1—C1	2.115 (5)
Sn1—C2	2.122 (5)
Sn1—C3	2.139 (4)
Sn1—O1*w*	2.472 (3)
Sn1—Cl1	2.485 (1)

**Table:** 

C1—Sn1—C2	120.2 (2)
C1—Sn1—C3	121.1 (2)
C2—Sn1—C3	116.0 (2)
O1*w*—Sn1—Cl1	176.1 (1)

**Fig. 1.**
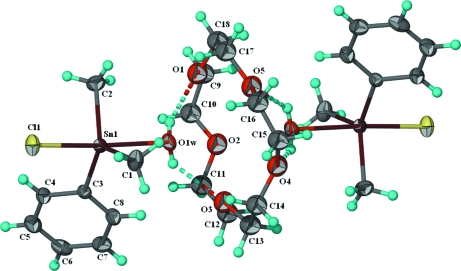
Molecular structure of (I); ellipsoids are drawn at the 30% probability level, and H atoms as spheres of arbitrary radius. The 15-crown-5 is disordered about a center of inversion, only one orientation is shown for reasons of clarity. Dashed lines denote the water···crown-ether hydrogen bonds. Unlabelled atoms are related by the symmetry operation -x + 2, -y + 1, -z + 2.

## supplementary crystallographic information

### Comment

Water-coordinated triorganotin salts form "outer-sphere coordination" complexes with crown ethers. In these, the water molecules interact with the crown ether through hydrogen bonds (Amini *et al.*, 1994; Chee *et al.*, 2003; Yap *et al.*, 1996). The refinement of such crystal structures present special difficulties when the odd-numbered crown ether is disordered about a center of inversion. The refinement of such crystal structures has been described in detail (Ng, 2005). The mixed-organo Sn^IV^ title compound, (I), has the tin atom in a *trans-*C_3_SnClO trigonal bipyramidal geometry, Fig. 1 & Table 1, in which the electronegative substituents occupy the axial sites. The coordinated water molecule forms two hydrogen bonds to the 15-crown-5, which is disordered about a center-of-inversion; Table 2.

### Experimental

ChlorodimethylphenylSn^IV^ was synthesized by the cleavage of dimethyldiphenyltin with hydrogen chloride in a methanol/carbon tetrachloride mixture at 283 K and was purified by distillation at 363 K/1 Torr. ChlorodimethylphenylSn^IV^ (0.26 g, 1 mmol) and 15-crown-5 (0.22 g, 1 mmol) were dissolved in ethanol (20 ml). The co-crystal (I), m.p. 347–349 K, separated when the solvent was allowed to evaporate. ^1^H-NMR in CDCl_3_: 0.90 (CH_3_), 1.60 (H_2_O), 3.72 (CH_2_), 7.27–7.70 (C_6_H_5_) p.p.m. ^119^Sn NMR 115.7 p.p.m. ^2^*J*(^119^Sn–^1^H) = 60 Hz.

### Refinement

The 15-crown-5 lies about a center of inversion and the molecule was refined as a 15-atom species of half site-occupancy. All 1,2-related atoms were restrained to 1.45±0.01 Å and 1,3-related atoms to 2.35±0.01 Å. The displacement factors of the O atoms were restrained to be equal, as were those of the C atoms.

The carbon-bound H atoms were placed in calculated positions with C–H 0.93 to 0.97 Å, and with *U*_iso_(H) 1.2–1.5*U*_eq_(C), and were included in the refinement in the riding-model approximation. The water H-atoms were placed in chemically sensible positions on the basis of hydrogen bonds but were not refined [O–H 0.82 Å and *U*_iso_(H) 1.5*U*_eq_(O)].

The final difference Fourier map had a relatively large peak/deep hole in the vicinity of the crown ether.

### Crystal data

**Table:**

2[Sn(CH_3_)_2_(C_6_H_5_)Cl(H_2_O)]·C_10_H_20_O_5_	*F*_000_ = 784
*M**_r_* = 778.91	*D*_x_ = 1.526 Mg m^−^^3^
Monoclinic, *P*2_1_/*n*	Mo *K*α radiation λ = 0.71073 Å
Hall symbol: -P 2yn	Cell parameters from 13887 reflections
*a* = 9.8700 (3) Å	θ = 3.1–27.4º
*b* = 18.9814 (5) Å	µ = 1.67 mm^−^^1^
*c* = 9.8770 (3) Å	*T* = 295 (2) K
β = 113.636 (1)º	Block, colorless
*V* = 1695.2 (1) Å^3^	0.30 × 0.25 × 0.20 mm
*Z* = 2	

### Data collection

**Table:**

Rigaku R-AXIS RAPID diffractometer	3869 independent reflections
Radiation source: fine-focus sealed tube	3433 reflections with *I* > 2σ(*I*)
Monochromator: graphite	*R*_int_ = 0.023
Detector resolution: 10.0 pixels mm^-1^	θ_max_ = 27.4º
*T* = 295(2) K	θ_min_ = 3.1º
ω scans	*h* = −10→12
Absorption correction: multi-scan(ABSCOR; Higashi, 1995)	*k* = −24→24
*T*_min_ = 0.534, *T*_max_ = 0.732	*l* = −12→12
16379 measured reflections	

### Refinement

**Table:**

Refinement on *F*^2^	Secondary atom site location: difference Fourier map
Least-squares matrix: full	Hydrogen site location: inferred from neighbouring sites
*R*[*F*^2^ > 2σ(*F*^2^)] = 0.042	H-atom parameters constrained
*wR*(*F*^2^) = 0.128	*w* = 1/[σ^2^(*F*_o_^2^) + (0.0816*P*)^2^ + 1.7228*P*] where *P* = (*F*_o_^2^ + 2*F*_c_^2^)/3
*S* = 1.06	(Δ/σ)_max_ = 0.001
3869 reflections	Δρ_max_ = 1.29 e Å^−^^3^
159 parameters	Δρ_min_ = −1.14 e Å^−^^3^
30 restraints	Extinction correction: none
Primary atom site location: structure-invariant direct methods	

### Fractional atomic coordinates and isotropic or equivalent isotropic displacement parameters (Å^2^)

**Table:**

	*x*	*y*	*z*	*U*_iso_*/*U*_eq_	Occ. (<1)
Sn1	0.55993 (3)	0.385283 (13)	0.73184 (3)	0.04272 (13)	
Cl1	0.31360 (13)	0.32890 (8)	0.60646 (14)	0.0700 (3)	
O1w	0.7986 (3)	0.44517 (17)	0.8682 (4)	0.0634 (8)	
H1w	0.7920	0.4866	0.8431	0.095*	
H2w	0.8636	0.4260	0.8495	0.095*	
C1	0.6070 (6)	0.3380 (3)	0.9397 (6)	0.0719 (14)	
H1A	0.6438	0.3730	1.0159	0.108*	
H1B	0.6802	0.3019	0.9571	0.108*	
H1C	0.5184	0.3175	0.9406	0.108*	
C2	0.4774 (6)	0.4899 (3)	0.6902 (7)	0.0696 (13)	
H2A	0.5512	0.5202	0.6813	0.104*	
H2B	0.4537	0.5056	0.7704	0.104*	
H2C	0.3901	0.4910	0.6000	0.104*	
C3	0.6535 (4)	0.34520 (18)	0.5860 (4)	0.0448 (8)	
C4	0.5616 (6)	0.3317 (2)	0.4393 (5)	0.0584 (10)	
H4	0.4599	0.3374	0.4072	0.070*	
C5	0.6219 (7)	0.3095 (3)	0.3396 (6)	0.0741 (14)	
H5	0.5592	0.2999	0.2422	0.089*	
C6	0.7687 (7)	0.3019 (3)	0.3827 (6)	0.0749 (14)	
H6	0.8073	0.2881	0.3150	0.090*	
C7	0.8608 (6)	0.3144 (3)	0.5261 (7)	0.0719 (13)	
H7	0.9623	0.3087	0.5563	0.086*	
C8	0.8038 (5)	0.3356 (3)	0.6281 (5)	0.0602 (11)	
H8	0.8678	0.3435	0.7259	0.072*	
O1	0.8394 (11)	0.5945 (5)	0.8704 (10)	0.0982 (12)	0.50
O2	1.0758 (10)	0.5345 (4)	0.8227 (10)	0.0982 (12)	0.50
O3	1.0975 (10)	0.4055 (5)	0.9549 (9)	0.0982 (12)	0.50
O4	1.1086 (10)	0.4378 (5)	1.2332 (10)	0.0982 (12)	0.50
O5	0.9190 (10)	0.5479 (5)	1.1592 (10)	0.0982 (12)	0.50
C9	0.9218 (17)	0.6312 (6)	0.8003 (17)	0.0922 (11)	0.50
H9A	0.8597	0.6661	0.7317	0.111*	0.50
H9B	1.0062	0.6550	0.8739	0.111*	0.50
C10	0.9708 (14)	0.5796 (6)	0.7224 (12)	0.0922 (11)	0.50
H10A	0.8869	0.5523	0.6574	0.111*	0.50
H10B	1.0140	0.6034	0.6622	0.111*	0.50
C11	1.1043 (16)	0.4717 (5)	0.7580 (12)	0.0922 (11)	0.50
H11A	1.1598	0.4829	0.6990	0.111*	0.50
H11B	1.0118	0.4498	0.6943	0.111*	0.50
C12	1.1889 (15)	0.4240 (7)	0.8775 (15)	0.0922 (11)	0.50
H12A	1.2159	0.3821	0.8379	0.111*	0.50
H12B	1.2788	0.4469	0.9443	0.111*	0.50
C13	1.1716 (15)	0.3633 (6)	1.0805 (11)	0.0922 (11)	0.50
H13A	1.2727	0.3797	1.1304	0.111*	0.50
H13B	1.1744	0.3150	1.0497	0.111*	0.50
C14	1.1003 (19)	0.3657 (6)	1.1805 (15)	0.0922 (11)	0.50
H14A	1.1492	0.3340	1.2629	0.111*	0.50
H14B	0.9978	0.3512	1.1308	0.111*	0.50
C15	1.0198 (14)	0.4487 (6)	1.3105 (12)	0.0922 (11)	0.50
H15A	0.9783	0.4036	1.3200	0.111*	0.50
H15B	1.0831	0.4641	1.4094	0.111*	0.50
C16	0.9041 (13)	0.4965 (6)	1.2534 (15)	0.0922 (11)	0.50
H16A	0.8925	0.5199	1.3353	0.111*	0.50
H16B	0.8138	0.4704	1.1999	0.111*	0.50
C17	0.7917 (15)	0.5900 (8)	1.0850 (13)	0.0922 (11)	0.50
H17A	0.7720	0.6189	1.1561	0.111*	0.50
H17B	0.7066	0.5599	1.0357	0.111*	0.50
C18	0.8149 (16)	0.6343 (6)	0.9783 (12)	0.0922 (11)	0.50
H18A	0.7289	0.6641	0.9308	0.111*	0.50
H18B	0.8995	0.6645	1.0283	0.111*	0.50

### Atomic displacement parameters (Å^2^)

**Table:**

	*U*^11^	*U*^22^	*U*^33^	*U*^12^	*U*^13^	*U*^23^
Sn1	0.04543 (19)	0.04251 (18)	0.03998 (18)	−0.00211 (9)	0.01686 (13)	0.00193 (8)
Cl1	0.0538 (6)	0.0880 (8)	0.0622 (7)	−0.0244 (6)	0.0171 (5)	−0.0039 (6)
O1w	0.0542 (16)	0.0637 (18)	0.068 (2)	−0.0146 (14)	0.0198 (14)	−0.0190 (15)
C1	0.072 (3)	0.082 (3)	0.052 (3)	−0.010 (3)	0.015 (2)	0.019 (2)
C2	0.067 (3)	0.053 (2)	0.087 (4)	0.007 (2)	0.029 (3)	0.007 (2)
C3	0.0526 (19)	0.0369 (16)	0.0439 (19)	−0.0036 (15)	0.0183 (16)	0.0006 (14)
C4	0.066 (3)	0.062 (2)	0.041 (2)	0.004 (2)	0.0158 (18)	−0.0013 (17)
C5	0.097 (4)	0.076 (3)	0.049 (3)	0.005 (3)	0.028 (3)	−0.006 (2)
C6	0.110 (4)	0.061 (3)	0.074 (3)	0.003 (3)	0.058 (3)	−0.008 (2)
C7	0.071 (3)	0.071 (3)	0.086 (4)	0.006 (2)	0.044 (3)	−0.006 (3)
C8	0.058 (2)	0.069 (3)	0.055 (2)	−0.006 (2)	0.024 (2)	−0.013 (2)
O1	0.094 (3)	0.106 (3)	0.088 (3)	0.004 (3)	0.031 (2)	0.003 (2)
O2	0.094 (3)	0.106 (3)	0.088 (3)	0.004 (3)	0.031 (2)	0.003 (2)
O3	0.094 (3)	0.106 (3)	0.088 (3)	0.004 (3)	0.031 (2)	0.003 (2)
O4	0.094 (3)	0.106 (3)	0.088 (3)	0.004 (3)	0.031 (2)	0.003 (2)
O5	0.094 (3)	0.106 (3)	0.088 (3)	0.004 (3)	0.031 (2)	0.003 (2)
C9	0.092 (2)	0.104 (3)	0.090 (3)	0.007 (2)	0.046 (2)	−0.005 (2)
C10	0.092 (2)	0.104 (3)	0.090 (3)	0.007 (2)	0.046 (2)	−0.005 (2)
C11	0.092 (2)	0.104 (3)	0.090 (3)	0.007 (2)	0.046 (2)	−0.005 (2)
C12	0.092 (2)	0.104 (3)	0.090 (3)	0.007 (2)	0.046 (2)	−0.005 (2)
C13	0.092 (2)	0.104 (3)	0.090 (3)	0.007 (2)	0.046 (2)	−0.005 (2)
C14	0.092 (2)	0.104 (3)	0.090 (3)	0.007 (2)	0.046 (2)	−0.005 (2)
C15	0.092 (2)	0.104 (3)	0.090 (3)	0.007 (2)	0.046 (2)	−0.005 (2)
C16	0.092 (2)	0.104 (3)	0.090 (3)	0.007 (2)	0.046 (2)	−0.005 (2)
C17	0.092 (2)	0.104 (3)	0.090 (3)	0.007 (2)	0.046 (2)	−0.005 (2)
C18	0.092 (2)	0.104 (3)	0.090 (3)	0.007 (2)	0.046 (2)	−0.005 (2)

### Geometric parameters (Å, °)

**Table:**

Sn1—C1	2.115 (5)	O4—C15	1.389 (8)
Sn1—C2	2.122 (5)	O4—C14	1.454 (9)
Sn1—C3	2.139 (4)	O5—C16	1.397 (8)
Sn1—O1w	2.472 (3)	O5—C17	1.420 (9)
Sn1—Cl1	2.485 (1)	C9—C10	1.442 (9)
O1w—H1w	0.8200	C9—H9A	0.9700
O1w—H2w	0.8200	C9—H9B	0.9700
C1—H1A	0.9600	C10—H10A	0.9700
C1—H1B	0.9600	C10—H10B	0.9700
C1—H1C	0.9600	C11—C12	1.455 (9)
C2—H2A	0.9600	C11—H11A	0.9700
C2—H2B	0.9600	C11—H11B	0.9700
C2—H2C	0.9600	C12—H12A	0.9700
C3—C8	1.383 (6)	C12—H12B	0.9700
C3—C4	1.390 (6)	C13—C14	1.425 (8)
C4—C5	1.404 (7)	C13—H13A	0.9700
C4—H4	0.9300	C13—H13B	0.9700
C5—C6	1.345 (8)	C14—H14A	0.9700
C5—H5	0.9300	C14—H14B	0.9700
C6—C7	1.362 (8)	C15—C16	1.388 (8)
C6—H6	0.9300	C15—H15A	0.9700
C7—C8	1.396 (7)	C15—H15B	0.9700
C7—H7	0.9300	C16—H16A	0.9700
C8—H8	0.9300	C16—H16B	0.9700
O1—C18	1.404 (9)	C17—C18	1.437 (8)
O1—C9	1.442 (9)	C17—H17A	0.9700
O2—C10	1.402 (8)	C17—H17B	0.9700
O2—C11	1.433 (9)	C18—H18A	0.9700
O3—C13	1.410 (9)	C18—H18B	0.9700
O3—C12	1.441 (9)		
			
C1—Sn1—C2	120.2 (2)	O2—C10—C9	110.4 (8)
C1—Sn1—C3	121.1 (2)	O2—C10—H10A	109.6
C2—Sn1—C3	116.0 (2)	C9—C10—H10A	109.6
C1—Sn1—O1w	82.9 (2)	O2—C10—H10B	109.6
C2—Sn1—O1w	83.3 (2)	C9—C10—H10B	109.6
C3—Sn1—O1w	87.5 (1)	H10A—C10—H10B	108.1
C1—Sn1—Cl1	95.2 (2)	O2—C11—C12	107.9 (8)
C2—Sn1—Cl1	94.8 (2)	O2—C11—H11A	110.1
C3—Sn1—Cl1	96.4 (1)	C12—C11—H11A	110.1
O1w—Sn1—Cl1	176.1 (1)	O2—C11—H11B	110.1
Sn1—O1w—H1w	109.5	C12—C11—H11B	110.1
Sn1—O1w—H2w	109.5	H11A—C11—H11B	108.4
H1w—O1w—H2w	109.5	O3—C12—C11	107.7 (8)
Sn1—C1—H1A	109.5	O3—C12—H12A	110.2
Sn1—C1—H1B	109.5	C11—C12—H12A	110.2
H1A—C1—H1B	109.5	O3—C12—H12B	110.2
Sn1—C1—H1C	109.5	C11—C12—H12B	110.2
H1A—C1—H1C	109.5	H12A—C12—H12B	108.5
H1B—C1—H1C	109.5	O3—C13—C14	111.5 (8)
Sn1—C2—H2A	109.5	O3—C13—H13A	109.3
Sn1—C2—H2B	109.5	C14—C13—H13A	109.3
H2A—C2—H2B	109.5	O3—C13—H13B	109.3
Sn1—C2—H2C	109.5	C14—C13—H13B	109.3
H2A—C2—H2C	109.5	H13A—C13—H13B	108.0
H2B—C2—H2C	109.5	C13—C14—O4	107.7 (8)
C8—C3—C4	117.5 (4)	C13—C14—H14A	110.2
C8—C3—Sn1	123.0 (3)	O4—C14—H14A	110.2
C4—C3—Sn1	119.3 (3)	C13—C14—H14B	110.2
C3—C4—C5	120.2 (5)	O4—C14—H14B	110.2
C3—C4—H4	119.9	H14A—C14—H14B	108.5
C5—C4—H4	119.9	C16—C15—O4	118.2 (8)
C6—C5—C4	121.1 (5)	C16—C15—H15A	107.8
C6—C5—H5	119.5	O4—C15—H15A	107.8
C4—C5—H5	119.5	C16—C15—H15B	107.8
C5—C6—C7	119.7 (5)	O4—C15—H15B	107.8
C5—C6—H6	120.1	H15A—C15—H15B	107.1
C7—C6—H6	120.1	C15—C16—O5	116.0 (8)
C6—C7—C8	120.4 (5)	C15—C16—H16A	108.3
C6—C7—H7	119.8	O5—C16—H16A	108.3
C8—C7—H7	119.8	C15—C16—H16B	108.3
C3—C8—C7	121.0 (5)	O5—C16—H16B	108.3
C3—C8—H8	119.5	H16A—C16—H16B	107.4
C7—C8—H8	119.5	O5—C17—C18	109.8 (8)
C18—O1—C9	114.0 (8)	O5—C17—H17A	109.7
C10—O2—C11	114.5 (8)	C18—C17—H17A	109.7
C13—O3—C12	113.0 (7)	O5—C17—H17B	109.7
C15—O4—C14	112.3 (8)	C18—C17—H17B	109.7
C16—O5—C17	115.4 (8)	H17A—C17—H17B	108.2
C10—C9—O1	107.7 (8)	O1—C18—C17	111.6 (8)
C10—C9—H9A	110.2	O1—C18—H18A	109.3
O1—C9—H9A	110.2	C17—C18—H18A	109.3
C10—C9—H9B	110.2	O1—C18—H18B	109.3
O1—C9—H9B	110.2	C17—C18—H18B	109.3
H9A—C9—H9B	108.5	H18A—C18—H18B	108.0
			
C1—Sn1—C3—C8	−54.1 (4)	C18—O1—C9—C10	165.5 (11)
C2—Sn1—C3—C8	107.3 (4)	C11—O2—C10—C9	165.4 (11)
O1w—Sn1—C3—C8	26.0 (4)	O1—C9—C10—O2	−67.6 (15)
Cl1—Sn1—C3—C8	−154.1 (3)	C10—O2—C11—C12	−167.9 (11)
C1—Sn1—C3—C4	129.8 (3)	C13—O3—C12—C11	−175.6 (11)
C2—Sn1—C3—C4	−68.8 (4)	O2—C11—C12—O3	63.6 (15)
O1w—Sn1—C3—C4	−150.1 (3)	C12—O3—C13—C14	160.1 (12)
Cl1—Sn1—C3—C4	29.7 (3)	O3—C13—C14—O4	−63.5 (16)
C8—C3—C4—C5	−0.2 (7)	C15—O4—C14—C13	169.2 (11)
Sn1—C3—C4—C5	176.1 (4)	C14—O4—C15—C16	−117.0 (15)
C3—C4—C5—C6	−1.1 (8)	O4—C15—C16—O5	−24 (2)
C4—C5—C6—C7	1.4 (9)	C17—O5—C16—C15	171.3 (13)
C5—C6—C7—C8	−0.6 (9)	C16—O5—C17—C18	−172.7 (12)
C4—C3—C8—C7	1.1 (7)	C9—O1—C18—C17	−155.4 (12)
Sn1—C3—C8—C7	−175.1 (4)	O5—C17—C18—O1	61.1 (16)
C6—C7—C8—C3	−0.7 (8)		

### Hydrogen-bond geometry (Å, °)

**Table:**

*D*—H···*A*	*D*—H	H···*A*	*D*···*A*	*D*—H···*A*
O1w—H1w···O1	0.82	2.09	2.86 (1)	156
O1w—H1w···O4^i^	0.82	2.05	2.74 (1)	143
O1w—H2w···O3	0.82	2.15	2.82 (1)	139
O1w—H2w···O5^i^	0.82	2.24	2.91 (1)	139

Symmetry codes: (i) −*x*+2, −*y*+1, −*z*+2.
